# Bayesian Sequential Monitoring of Single-Arm Trials: A Comparison of Futility Rules Based on Binary Data

**DOI:** 10.3390/ijerph18168816

**Published:** 2021-08-20

**Authors:** Valeria Sambucini

**Affiliations:** Dipartimento di Scienze Statistiche, Sapienza University of Rome, Piazzale Aldo Moro 5, 00185 Rome, Italy; valeria.sambucini@uniroma1.it

**Keywords:** Bayesian monitoring, futility rules, interim analysis, posterior and predictive probabilities, stopping boundaries

## Abstract

In clinical trials, futility rules are widely used to monitor the study while it is in progress, with the aim of ensuring early termination if the experimental treatment is unlikely to provide the desired level of efficacy. In this paper, we focus on Bayesian strategies to perform interim analyses in single-arm trials based on a binary response variable. Designs that exploit both posterior and predictive probabilities are described and a slight modification of the futility rules is introduced when a fixed historical response rate is used, in order to add uncertainty in the efficacy probability of the standard treatment through the use of prior distributions. The stopping boundaries of the designs are compared under the same trial settings and simulation studies are performed to evaluate the operating characteristics when analogous procedures are used to calibrate the probability cut-offs of the different decision rules.

## 1. Introduction

In clinical trials, the implementation of data monitoring for early termination represents a frequently used strategy. In many trials, participants are followed for a relatively long period and, therefore, it may be desirable to conduct interim analyses during the course of the trial with the aim of early stopping the study if there is convincing evidence of benefit or harm. The Bayesian approach is particularly suited to this experimental context, since it naturally entails sequential updating of the interim decision rules as data accumulate.

Let us focus on single-arm designs that are typically used in phase II trials, whose primary goal is not to provide definitive evidence of drug efficacy, but to avoid further investigations for unpromising drugs. In this early phase, ethical concerns make it especially important to establish convincing futility stopping rules to reduce the number of patients who receive ineffective treatments. A binary efficacy variable is typically considered and the response rate of the experimental treatment is usually compared with a constant target value that should ideally represent the response rate for the standard of care therapy. Generally, this target value is fixed by exploiting historical information about the efficacy of the standard treatment that is typically available.

Under a Bayesian framework, monitoring strategies of single-arm phase II trials are typically based on either posterior probabilities or predictive probabilities [1]. Thall and Simon [2] proposed a Bayesian procedure that continually evaluates, as data accumulate, the posterior probability that the experimental treatment is superior to the standard one, until reaching a maximum planned sample size *N*. At any interim stage, given the current data, the futility rule determines the termination of the trial if the posterior probability of interest is lower than a fixed threshold. An important feature of the design is that it avoids the specification of a fixed target to evaluate the efficacy of the experimental drug, while accounting for the uncertainty in the response rate of the standard agent by the use of prior distributions. This makes it possible to incorporate in a more realistic way pre-experimental knowledge about the standard treatment [3]. The design proposed by Thall and Simon [2] has been extended to accommodate the monitoring of both efficacy and safety endpoints [4,5,6]. Zhou et al. [7] presented a unified approach to construct a Bayesian optimal design for phase II trials (BOP2) based on posterior probabilities, that can handle binary and more complicated endpoints through the use of a Dirichlet-multinomial model. Differently from the proposal of Thall and Simon [2], the BOP2 design does not exploit prior distributions to introduce uncertainty in the historical response rate. However, a merit of the design is that its futility rule compares the posterior probability that the response rate of the experimental treatment exceeds the target level with a threshold that varies as a function of n/N, where *n* is the current sample size. This allows to have a more relaxed stopping rule at the initial stages of the trial, when the accumulated information is limited, in order to avoid early stopping of the study on the basis of fortuitously negative results. More recently, simulation tools have been exploited to compare the use of alternative probability boundaries with different shapes as functions of the interim sample size [8].

For interim monitoring, Bayesian methods based on predictive probabilities are also widely used in practice [9]. The idea is to evaluate the chance of having a desired outcome at the scheduled end of the trial conditional on the observed interim data [10]. Lee and Liu [11] described how to implement predictive decision rules in single-arm phase II trials based on a binary endpoint. The condition to establish if the experimental treatment can be declared successful at the conclusion of the trial is based on the posterior probability that its response rate exceeds a fixed target level. At any interim stage, it is possible to obtain the predictive probability that this condition is attained by enumerating all possible future outcomes. According to the futility rule, the trial is stopped for lack of efficacy if this predictive probability is below a threshold of interest. The predictive probability monitoring is considered conceptually appealing because it takes into account the uncertainty in future data [12]: it mimics the decision-making process of claiming the drug promising or non-promising by projecting the result to the end of the trial [13]. This very flexible approach has been also applied to more complex trial settings, such as randomized phase II trials [14], platform studies [15], trials that simultaneously monitor efficacy and safety [16], and studies based on time-to-event endpoints [17] or longitudinal outcomes [18].

In this paper, we focus on Bayesian single-arm designs based on both posterior and predictive probabilities. More specifically, we aim at comparing the phase II design of Thall and Simon [2] with slightly modified versions of the designs due to Zhou et al. [7] and Lee and Liu [11] that account for the uncertainty in the response rate of the standard treatment. All three designs allow to enumerate the stopping boundaries of the futility rules before the trial starts. For each current sample size of interest, these boundaries are provided in terms of the maximum number of responses that, if observed, leads to the termination of the study for lack of efficacy. This common characteristic makes the designs particularly easy to implement in practice, because it avoids the need to implement Bayesian computation at interim analyses during the trial. We compare the stopping boundaries of the three designs under the same trial settings and using analogous procedures to calibrate the probability cut-offs of the different decision rules. The frequentist performance of the designs have been also evaluated through simulations.

The outline of the paper is as follows. Section 2 provides some preliminaries on the Bayesian problem setting when the focus is on of a single-arm trial based on a binary endpoint. In Section 3 and Section 4, we review the futility monitoring rules based on posterior and predictive probabilities, respectively. We also introduce modified versions of the designs due to Zhou et al. [7] and Lee and Liu [11], that exploit prior distributions of the probability efficacy of the standard treatment. The calibration of the probability thresholds is also discussed. In Section 5 we present the results of simulation studies that evaluate and compare the operating characteristics of the Bayesian designs. Finally, Section 6 contains a conclusive discussion.

## 2. Bayesian Problem Settings

Let us consider a single-arm phase II trial based on a binary endpoint that represents the efficacy of an experimental treatment, *E*, and assume that a standard treatment, *S*, exists for the disease under study. The parameter of interest of the trial is the response rate of *E*, denoted by $p_{E}$. Due to the non-comparative nature of the study, $p_{E}$ is typically compared with a fixed target value $p_{S}^{*}$, usually obtained by exploiting historical data on the efficacy probability of *S*. In practice, $p_{S}^{*}$ is typically set equal to the historical estimate of the response rate of the standard therapy or equal to the estimate plus a minimum clinically meaningful improvement. Then, the new treatment is considered sufficiently promising if $p_{E}$ exceeds $p_{S}^{*}$.

Let *N* be the maximum sample size planned for the entire study. We assume that the number of responses in the current *n* (n≤N) patients at a certain interim time, *X*, follows a binomial distribution with parameters *n* and $p_{E}$. We denote by beta(·;α,β), and Beta(·;α,β) the probability density function and the cumulative distribution function of a beta distribution with parameters α and β, respectively. By introducing a beta prior distribution for $p_{E}$, $\pi \left(p_{E}\right)=\mathrm{beta}\left(p_{E};\alpha_{E},\beta_{E}\right)$, from standard Bayesian conjugate analysis it follows that the corresponding posterior distribution is still a beta density,

(1) $$\begin{array}{c} \pi \left(p_{E}|x,n\right)=\mathrm{beta}\left(p_{E};\alpha_{E}+x,\beta_{E}+n-x\right). \end{array}$$

Therefore, the posterior probability that $p_{E}$ exceeds the target $p_{S}^{*}$ can be easily computed as

(2) $$\begin{array}{c} 1-\mathrm{Beta}(p_{S}^{*};\alpha_{E}+x,\beta_{E}+n-x). \end{array}$$

Aside from computational convenience, the beta prior distribution is typically employed because of its capability of assuming a wide variety of shapes reflecting various degrees of prior belief. In general terms, in order to elicit different kinds of available information or to represent reasonable skeptical or enthusiastic opinions regarding a success probability *p*, the hyperparameters α and β of a beta prior are often expressed in terms of (i) a *measure of central location* and (ii) a parameter representing the *prior sample size*. For instance, by setting

$$\begin{array}{c} \alpha =n^{prior}p^{prior}+1\;\mathrm{and}\;\beta =n^{prior}\left(1-p^{prior}\right)+1, \end{array}$$

we obtain a prior density with mode at $p^{prior}$ and prior sample size $n^{prior}$, that reflects the dispersion of the distribution around its mode. The larger the value of $n^{prior}$, the more concentrated is the beta prior [19]. A similar and alternative way of proceeding is to choose the prior mean as the measure of centrality of interest, $p^{prior}$. In this latter case, the hyperparameters are fixed as $\alpha =n^{prior}p^{prior}$ and $\beta =n^{prior}\left(1-p^{prior}\right)$.

## 3. Futility Rules Based on Posterior Probabilities

### 3.1. The Design of Thall and Simon

Thall and Simon [2] proposed a Bayesian single arm design for phase II trials, where at each interim look the futility rule is based on the posterior probability that the experimental treatment is more effective than the standard one. In the original proposal, data are monitored continuously until the maximum planned sample size is reached, but actually the design can be implemented by using cohorts of different sizes.

Let us denote by $p_{S}$ the unknown response rate of the standard treatment. Instead of using a pre-specified target value $p_{S}^{*}$ in order to establish if the treatment *E* can be considered sufficiently promising, the authors fully exploit the Bayesian approach and treat both $p_{E}$ and $p_{S}$ as random variables. Thus, we consider two independent prior distributions,

$$\begin{array}{c} \pi \left(p_{E}\right)=\mathrm{beta}\left(p_{E};\alpha_{E},\beta_{E}\right)\;\mathrm{and}\;\pi \left(p_{S}\right)=\mathrm{beta}\left(p_{S};\alpha_{S},\beta_{S}\right). \end{array}$$

The prior $\pi (p_{S})$ is constructed as an informative distribution based on historical data about *S*, whose weight can be discounted by using suitable procedures that allow to enlarge the prior variance [20,21]. Alternative strategies to build informative prior distributions for a response rate in phase II trials are provided in the literature [22,23,24]. For $p_{E}$, instead, it could be reasonable to elicit a non-informative or a very diffuse prior density, since little pre-experimental information is generally available about the novel therapy. Many authors suggest to center this prior density at a value $p_{E}^{prior}$ considered the most likely, while fixing the prior sample size equal to one [8,19,25,26]. As stated by Tan and Machin [26] “such a prior distribution is sufficiently vague to allow for the possibility that $p_{E}$ may take any value in the range (0, 1), although its most likely value is $p_{E}^{prior}$”.

Then, given *x* responses observed out of *n* current patients treated with the experimental agent, the joint posterior distribution of $\left(p_{E},\;p_{S}\right)$ is

(3) $$\begin{array}{c} \pi \left(p_{E},p_{S}|x,n\right)\propto \mathrm{beta}\left(p_{E};\alpha_{E}+x,\beta_{E}+n-x\right)\mathrm{beta}\left(p_{S};\alpha_{S},\beta_{S}\right). \end{array}$$

The experimental drug is considered sufficiently promising if $p_{E}>p_{S}+\delta$, where δ denotes the minimally acceptable increment in the efficacy rate for *E* compared with *S*. Therefore, the posterior probability that the experimental treatment is worthy of further evaluation can be computed as

(4) $$\begin{array}{ccc} & & \Pi_{E,S}\left(p_{E}>p_{S}+\delta |x,n\right)=\\ & & \int_{0}^{1-\delta }\left[1-\mathrm{Beta}(p_{S}+\delta ;\alpha_{E}+x,\beta_{E}+n-x)\right]\mathrm{beta}\left(p_{S};\alpha_{S},\beta_{S}\right)dp_{S}, \end{array}$$

where $\Pi_{E,S}$ indicates the probability measure corresponding to the posterior distribution in (3). The integral in (4) can be evaluated numerically. The use of a prior distribution for $p_{S}$ allows to incorporate uncertainty in the historical response rate of the standard agent and, if no uncertainty is introduced by setting $\pi (p_{S})$ equal to a degenerate density at the target $p_{S}^{*}$, the posterior quantity in (4) is simply reduced to (2) for δ=0.

The futility stopping rule consists in terminating the trial and declaring the experimental drug not sufficiently promising if

(5) $$\begin{array}{c} \Pi_{E,S}\left(p_{E}>p_{S}+\delta |x,n\right)\leq C, \end{array}$$

where *C* is a pre-specified probability threshold. Thall and Simon [2] suggest to set *C* as a small value, so that the criterion in (5) allows to terminate the study if, given the current data, it is very unlikely that the experimental treatment has superior efficacy over the standard one. However, regulators currently require the attainment of targeted frequentist operating characteristics to approve Bayesian designs, and simulations are commonly used to adjust tuning parameters to satisfy pre-specified constraints on the type I error probability [27].

In our setting, and under the hypothesis testing framework, an appropriate null hypothesis $H_{0}$ specifies values of the parameters under which the novel treatment is considered not worthy of further evaluation, while the alternative $H_{1}$ specifies values of the parameters under which the treatment is considered sufficiently promising. Therefore, we have that $H_{0}:p_{E}\leq p_{S}+\delta$ and $H_{1}:p_{E}>p_{S}+\delta$. Of course, the rejection of $H_{0}$ corresponds to the continuation of the trial. As *C* increases, it becomes harder to reject the null hypothesis and the type I error rate decreases. Therefore, assuming a suitable scenario under $H_{0}$, *C* is typically calibrated through simulation techniques as the smallest value that controls the type I error probability at a desired level. For instance, let us consider a trial with N=40, δ=0.1 and interim analyses conducted continuously after the first $N_{min}=10$ patients have been treated. Suppose that historical data indicate 0.4 as the estimate of the response rate of the standard treatment and suggest that is highly feasible that $p_{S}$ lies in the range [0.3, 0.5]. To take into account this prior knowledge when eliciting the beta prior distribution for $p_{S}$, we express the hyperparameters in terms of the prior mode and a suitable value for the prior sample size, as described in Section 2. Specifically, we set the mode equal to 0.4 and fix the prior sample size so that it is approximatively equal to 0.99 the prior probability assigned to the interval [0.3, 0.5]. This way of proceeding leads to the prior $\pi \left(p_{S}\right)=\mathrm{beta}\left(p_{S};\;63,\;94\right)$, based on a prior sample size equal to 155. The beta prior density for $p_{E}$ is assumed to be $\pi \left(p_{E}\right)=\mathrm{beta}\left(p_{E};\;1.4,\;1.6\right)$, which also has its mode at 0.4, but is much more diffuse being based on a prior sample size equal to 1. Then, for each element in a set of possible thresholds *C*, we simulate 100000 clinical trials assuming that the true $p_{E}$ is equal to 0.4 (scenario under $H_{0}$) and compute the type I error rate as the frequency of simulated trials that reach the maximum sample size and conclude rejecting the null hypothesis. The calibrated value of the threshold is the smallest element in the set that controls the error probability at the level 0.1. In the specific case considered we obtain the value 0.278.

Furthermore, since $\Pi_{E,S}\left(p_{E}>p_{S}+\delta |x,n\right)$ is a monotonic function of the number of current responses, it is possible to obtain the rejection regions of the design prior to the onset of the trial. Under the setup described above, the stopping boundaries are provided in Table 1.

In practice, the trial terminates for low efficacy if the number of responses after treating *n* patients is less than or equal to the corresponding boundary $r_{n}$.

### 3.2. The BOP2 Design

Zhou et al. [7] proposed a Bayesian optimal phase II (BOP2) design that is based on posterior probabilities and accommodates various types of endpoints. In the case of a binary efficacy endpoint, two essential differences from the design of Thall and Simon [2] are:

1. the experimental treatment is considered sufficiently promising if $p_{E}$ exceeds a constant target $p_{S}^{*}$;
2. the posterior probability of interest is compared with a threshold that varies with the interim sample size.

In other words, in line with the majority of phase II Bayesian designs, the BOP2 design does not introduce uncertainty on the efficacy rate of the standard therapy. Moreover, the design takes into account the weight of the current information in relation to the amount of future data. Let us recall that the decision rule in (5) depends on the constant cut-off *C*: the larger the cut-off is chosen, the more stringent is the criterion for going on with the trial. Instead of considering a fixed probability threshold, Zhou et al. [7] allow it to monotonically increase with the fraction of accumulated information, n/N. The idea is that, when *n* is small, a more relaxed stopping rule, based on smaller values of the probability threshold, is preferred to avoid terminating the trial for fortuitously negative results. As the trial proceeds and more data are accumulated, it is desirable to have a more stringent condition, based on larger values of the cut-off, in order to correctly identify ineffective treatments.

At a certain stage of the trial, when *x* responses have been observed out of *n* current patients, the futility rule of the BOP2 design consists in stopping the trial if

$$\begin{array}{c} \Pi_{E}\left(p_{E}>p_{S}^{*}|x,n\right)\leq C\left(n\right), \end{array}$$

where $\Pi_{E}$ indicates the probability measure corresponding to the posterior distribution in (1) and

(6) $$\begin{array}{c} C\left(n\right)=\lambda {\left(\frac{n}{N}\right)}^{\gamma }. \end{array}$$

The strictly positive tuning parameters, λ and γ, are selected by maximizing the power of the design while controlling the type I error rate at a certain level under suitable scenarios. As an alternative strategy, Zhou et al. [7] suggest to choose λ, γ and the maximum sample size *N* that yield the minimum expected sample size under $H_{0}$, while ensuring desirable levels for the type I and type II error rates. In this latter case, *N* is not fixed, but represents a design parameter to be optimized.

#### 3.2.1. Accounting for Uncertainty on $p_{S}$ in the BOP2 Design

In line with Thall and Simon [2], we modify the decision rule of the BOP2 design by introducing a prior distribution on $p_{S}$ that accounts for the uncertainty in the response rate of the standard treatment. The trial, therefore, terminates at the interim look if

(7) $$\begin{array}{c} \Pi_{E,S}\left(p_{E}>p_{S}+\delta |x,n\right)\leq C\left(n\right), \end{array}$$

where C(n) is the threshold in (6) whose tuning parameters can be calibrated by using the strategies described above. From now on, we will refer to the design based on the modified futility rule in (7) by using the acronym BOP2m, while the design of Thall and Simon [2] will be indicated as the TS design.

Let us consider again the trial continuously monitored with N=40, $N_{min}=10$, δ=0.1, $\pi \left(p_{S}\right)=\mathrm{beta}\left(p_{S};\;63,\;94\right)$, and $\pi \left(p_{E}\right)=\mathrm{beta}\left(p_{E};\;1.4,\;1.6\right)$. We calibrate the tuning parameters λ and γ through simulations by maximizing the statistical power when $p_{E}$ is equal to 0.6 (scenario under $H_{1}$), while ensuring that the type I error rate is smaller than or equal to the nominal level 0.1 when the true $p_{E}$ is 0.4 (scenario under $H_{0}$). More details about the grid search algorithm used to adjust the parameters will be provided in Section 5. The resulting calibrated values are λ=0.38 and γ=0.95 and we provide the corresponding stopping boundaries in Table 2.

**Figure 1:**
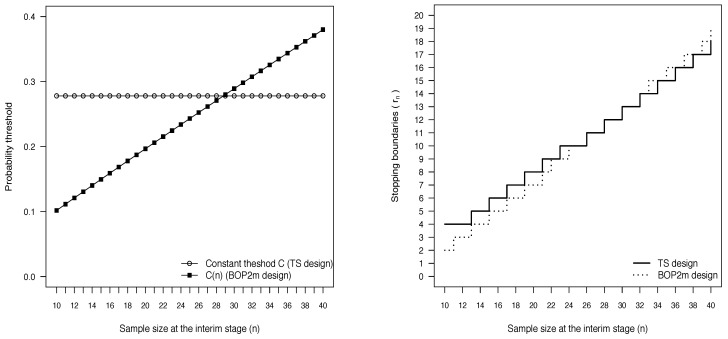
Behavior of the calibrated thresholds *C* and C(n) as a function of *n* (**left panel**) and stopping boundaries of the TS and BOP2m designs (**right panel**), when λ=0.38, γ=0.95, N=40, $N_{min}=10$, δ=0.1, $\pi \left(p_{S}\right)=\mathrm{beta}\left(p_{S};\;63,\;94\right)$, $\pi \left(p_{E}\right)=\mathrm{beta}\left(p_{E};\;1.4,\;1.6\right)$ and the nominal level for the type I error rate is 0.1.

In the left panel of Figure 1 we show the behavior of the calibrated thresholds *C* and C(n) as a function of the current sample size *n*. Differently from the threshold used in the TS design, that remains constant, the threshold of the BOP2m design increases as data accumulate: it is smaller than *C* for very low values of *n* and exceeds *C* when *n* approaches the maximum planned sample size. As a consequence, the BOP2m design makes it harder to terminate the trial at early stages of the study, while it is easier to stop at later stages, as it is evident looking at the right panel of Figure 1 where the stopping boundaries of both the designs are represented.

## 4. Futility Rules Based on Predictive Probabilities

### 4.1. The Design of Lee and Liu

In the Bayesian phase II design proposed by Lee and Liu [11], at any interim analysis, the futility rule is based on the evaluation of the predictive probability that the trial will show a conclusive result at the planned end of the study, given the observed data.

Given *x* responses observed in the current *n* patients, let *Y* be the random variable representing the number of responses out of the potential future N−n patients. It is well known that the posterior predictive distribution of *Y* is

(8) $$\begin{array}{c} m_{N}\left(y|x,n\right)=\text{beta-binom}\left(y;N-n,\alpha_{E}+x,\beta_{E}+n-x\right), \end{array}$$

for y=0,1,⋯,N−n. At the conclusion of the study, when the result Y=y will be available, the experimental treatment will be declared sufficiently promising if the following condition will be satisfied

$$\begin{array}{c} \Pi_{E}\left(p_{E}>p_{S}^{*}|x+y,N\right)>\theta_{T}, \end{array}$$

where $\theta_{T}$ is a pre-specified probability cut-off. However, at the interim look *Y* has not yet been observed and it is possible to exploit the posterior predictive distribution in (8) to calculate the probability of a positive conclusion should the trial be conducted to the maximum planned sample size, that is

(9) $$\begin{array}{c} PP=\sum_{y=0}^{N-n}m_{N}\left(y|x,n\right)I\left\{\Pi_{E}\left(p_{E}>p_{S}^{*}|x+y,N\right)>\theta_{T}\right\}, \end{array}$$

where I{·} denotes the indicator function. In practice, PP is obtained by summing the predictive probabilities of all the possible future outcomes that, given the accumulated information, will allow to declare that the experimental treatment is sufficiently promising at the end of the trial. The futility rule of the design is, therefore, to stop the trial and consider the experimental treatment not sufficiently good if PP is below a suitable fixed threshold $\theta_{L}$. A low value of PP in fact indicates that the new drug is likely to be declared ineffective by the end of the study. The thresholds $\theta_{T}$ and $\theta_{L}$ can be specified in order to optimize frequentist operating characteristics of the design.

Let us notice that this predictive design has two similarities with the BOP2: it does not account for uncertainty in the response rate of the standard treatment and it makes a compromise between the current information and the amount of future data. In fact, no prior distribution on $p_{S}$ is considered. Moreover, the decision rule based on predictive probability in (9) focuses on the expected results at the scheduled end of the trial and is affected by the number of remaining patients. More specifically, while in the BOP2 design the posterior quantity of interest is compared with a threshold that varies as a function of *n*, in the design of Lee and Liu the probability threshold $\theta_{L}$ is fixed, but the predictive probability PP varies as a function of the number of future patients and the futility rule generally results to be less stringent at the initial stages of the trial, when there is still a large number of patients to enrol.

#### 4.1.1. Accounting for Uncertainty on $p_{S}$ in the Design of Lee and Liu

Similarly to the BOP2 design, the predictive design of Lee and Liu [11] can also be modified to account for the uncertainty in the response rate of the standard therapy by introducing a beta prior distribution on $p_{S}$. Then, the decision rule stops accrual for futility if

(10) $$\begin{array}{c} PPm=\sum_{y=0}^{N-n}m_{N}\left(y|x,n\right)I\left\{\Pi_{E,S}\left(p_{E}>p_{S}+\delta |x+y,N\right)>\theta_{T}\right\}<\theta_{L}. \end{array}$$

Let us notice that PPm is reduced to PP if $p_{S}$ has a point mass distribution at $p_{S}^{*}$ and δ=0. From now on, the abbreviation LLm will be used to indicate the design based on the futility rule in (10).

**Figure 2:**
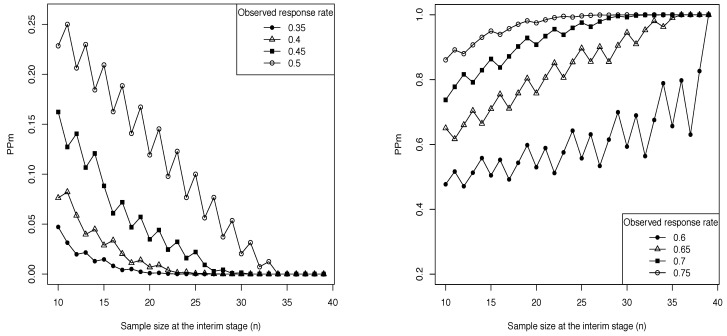
Behavior of PPm as a function of *n* for different values of the response rate assumed to be observed at the interim stage, when N=40, $N_{min}=10$, δ=0.1, $\pi \left(p_{S}\right)=\mathrm{beta}\left(p_{S};\;63,\;94\right)$, $\pi \left(p_{E}\right)=\mathrm{beta}\left(p_{E};\;1.4,\;1.6\right)$ and $\theta_{T}=0.8$.

It can be interesting to investigate how the predictive probability PPm is affected by the ratio between the amount of current information and the weight of future data, with the aim of better understand the behavior of the stopping boundaries of the LLm design as *n* increases. Let us refer again to the trial settings considered in the previous section: N=40, $N_{min}=10$, δ=0.1, $\pi \left(p_{S}\right)=\mathrm{beta}\left(p_{S};\;63,\;94\right)$ and $\pi \left(p_{E}\right)=\mathrm{beta}\left(p_{E};\;1.4,\;1.6\right)$. In practice, the experimental treatment is considered sufficiently promising if $p_{E}$ exceeds $p_{S}+0.1$, under the prior assumption that $p_{S}$ is centred on 0.4 and varies in the interval [0.3, 0.5] with high probability. Moreover, we assume that the study is monitored continuously end set the probability threshold $\theta_{T}$ equal to 0.8. We consider fixed values for the observed response rate obtained at the interim stage and, for each value of *n* between $N_{min}$ and N−1, we compute the corresponding predictive probability of interest. In the left panel of Figure 2, we show the behavior of PPm as a function of *n* for low values of the fixed response rate observed ad interim, while in the right panel higher values of the current response rate are considered. First of all, let us notice that the saw-toothed behavior of PPm in both the graphs is a consequence of the discrete nature of the predictive distribution of future data [28]. Moreover, as expected, the larger the response rate supposed to be observed out of *n* patients, the higher the predictive probability of a positive conclusion at the planned end of the trial. More importantly, we can note that in the left panel of Figure 2, even if there are some small fluctuations, the shape of PPm is basically decreasing. The fixed observed response rate can be obtained for different couples of the observed number of successes $x_{obs}$ and the current sample size *n*. For instance, when it is equal to 0.4, we have that PPm is equal to 0.0763, 0.0069, and 0.0000 for $x_{obs}/n$ equal to 4/10, 8/20 and 12/30, respectively. In practice, if *n* is small, there is still a high number of patients to be enrolled and, even if the observed response rate is low with respect to the design expectations, there is a non-negligible predictive probability that the study will conclude in favor of the experimental therapy. Instead, when *n* increases and the same response rate is obtained, the number of potential future patients decreases and it becomes very unlikely that the experimental treatment will be claimed sufficiently promising at the conclusion of the trial. The current information, in fact, has a stronger impact on the value of PPm as the future sample size decreases. The basically increasing behavior of PPm shown in the right panel of Figure 2 can be explained with an analogous reasoning. If the fixed response rate registered at the interim stage is high, as the number of future patients decreases, we have a stronger confidence that the superiority of the experimental treatment will be claimed at the scheduled end of the trial. This explain the behavior of PPm.

Furthermore, since PPm is a monotonic function of the number of current responses, it is possible to obtain the stopping boundaries of the LLm design before the beginning of the study. The smaller *n*, the lower the number of responses needed to let PPm reach the desired level $\theta_{L}$ to go on with the trial. Therefore, similarly to the BOP2m design, the predictive design typically makes it harder to stop the trial when the accumulated information at the interim stage is limited because based on a few patients. In order to have a fair comparison between the designs, under the trial settings previously considered, we use simulations to adjust the probability thresholds $\theta_{L}$ and $\theta_{T}$, so that the statistical power is maximized when $p_{E}$ is equal to 0.6 and the type I error rate is controlled at the level 0.1 when the true $p_{E}$ is 0.4. The resulting calibrated values are $\theta_{L}=0.011$ and $\theta_{T}=0.59$, and we provide the corresponding stopping boundaries in Table 3.

**Figure 3:**
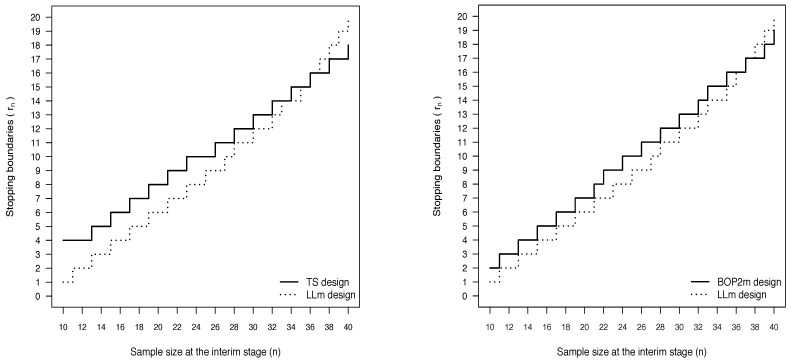
Comparison of stopping boundaries (TS vs. LLm (**left panel**) and BOP2m vs LLm (**right panel**)), when N=40, $N_{min}=10$, δ=0.1, $\pi \left(p_{S}\right)=\mathrm{beta}\left(p_{S};\;63,\;94\right)$, $\pi \left(p_{E}\right)=\mathrm{beta}\left(p_{E};\;1.4,\;1.6\right)$ and the nominal level for the type I error rate is 0.1.

In Figure 3, these stopping boundaries are compared with those of the TS and BOP2m designs provided in the previous sections and based on probability thresholds similarly calibrated. With respect to both the Bayesian designs based on posterior probabilities, the futility rules of the predictive design are less stringent at the initial stages of the trial. For small values of *n*, the LLm design requires lower values for the minimum number of responses necessary to let the trial proceed. On the contrary, when *n* is close to the maximum planned sample size, more responses are needed to avoid the termination of the study under the LLm design.

**Figure 4:**
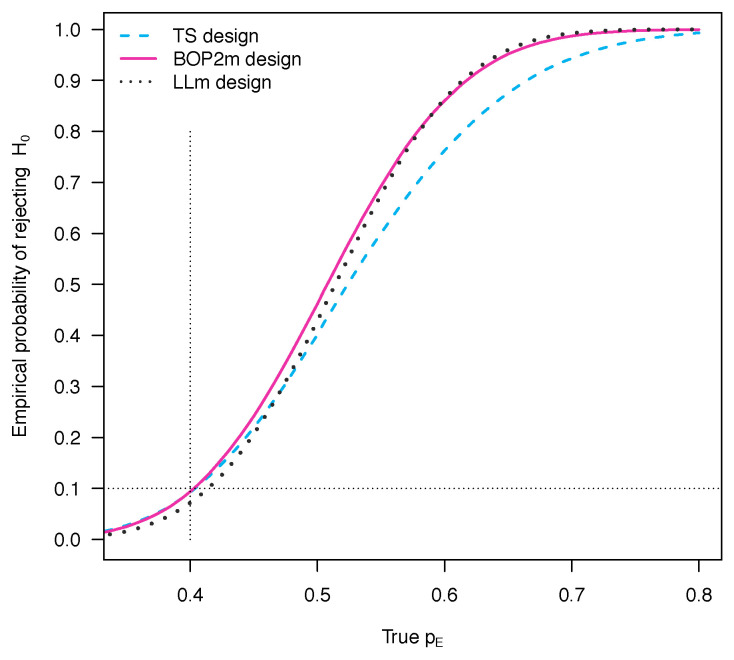
Behavior of the empirical probability of rejecting $H_{0}$ for different values of the true $p_{E}$, when N=40, $N_{min}=10$, δ=0.1, $\pi \left(p_{S}\right)=\mathrm{beta}\left(p_{S};\;63,\;94\right)$, and $\pi \left(p_{E}\right)=\mathrm{beta}\left(p_{E};\;1.4,\;1.6\right)$. The stopping boundaries used are provided in Section 3.1, Section 3.2.1 and Section 4.1.1 for the TS, the BOP2m and the LLm designs, respectively.

To compare the performance of the three Bayesian designs, we consider a dense set of values for $p_{E}$ in the interval [0.3, 0.8] and, for each value, we simulate 100,000 clinical trials to empirically evaluate the probability of rejecting $H_{0}$. Its behavior as a function of the true $p_{E}$ is shown in Figure 4 for each design. As expected, when $p_{E}$ is equal to 0.4, the probability of rejecting $H_{0}$ is below the level 0.1 for all the Bayesian designs. This is in fact a consequence of the calibration procedure of the probability cut-offs that ensures a type I error rate controlled at 0.1 under the null scenario where the response rate of the experimental drug is 0.4. When $p_{E}$ is higher than 0.4, the probability of rejecting $H_{0}$ corresponds to the statistical power, i.e., the probability of correctly concluding in favor of the experimental treatment. As $p_{E}$ varies, the BOP2m design and LLm design yield very similar power levels, which are substantially higher compared with those of the TS design. Thus, more power is gained by using futility rules that gradually become stringent as more patients are enrolled.

## 5. Comparison of the Operating Characteristics

In this section, we present the results of simulation studies aimed at evaluating and comparing the performance of the Bayesian futility rules previously described. More specifically, we consider the TS design and the modified versions of the BOP2 design and the predictive design due to Lee and Liu [11], presented in Section 3.2.1 and Section 4.1.1.

**Figure 5:**
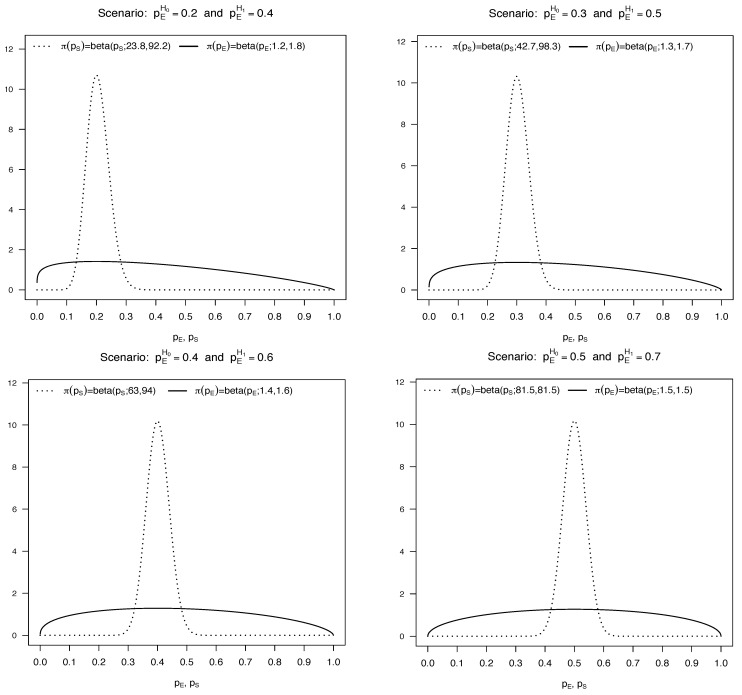
Beta prior distributions of $p_{E}$ and $p_{S}$ for each of the scenarios used in the simulation studies.

We assume that the first interim analysis is conducted after observing $N_{min}=10$ patients and, subsequently, data are monitored using cohorts of size *m* (with *m* equal to 1 or 5) until the maximum sample size *N* is reached (with *N* equal to 40 or 80). To calibrate the probability thresholds of the Bayesian designs, we specify different scenarios by identifying two values for $p_{E}$: one under the null hypothesis ($p_{E}^{H_{0}}$) and the other one under the alternative ($p_{E}^{H_{1}}$). In particular, we consider four possible values for $p_{E}^{H_{0}}$ and fix the corresponding $p_{E}^{H_{1}}$ equal to $p_{E}^{H_{0}}$ + 0.2. For each scenario, we elicit specific prior distributions for $p_{E}$ and $p_{S}$ obtained by expressing the hyperparameters in terms of the desired prior mode and a suitable prior sample size, as described in Section 2. The modes of both the beta prior densities are set equal to $p_{E}^{H_{0}}$, but their variability is quite different. In fact, the prior sample size of $\pi (p_{S})$ is selected to ensure that a large prior probability is assigned to a short interval centred at the prior mode. Specifically, we assign a prior probability about equal to 0.99 to the interval $\left(p_{E}^{H_{0}}-0.1,\;p_{E}^{H_{0}}-0.1\right)$. Instead, the prior sample size of $\pi (p_{E})$ is set equal to 1, in order of to obtain a flat density based on very weak information. We show the resulting prior distributions in Figure 5 for each of the four scenarios taken into account.

Let us recall that, when we simulate a high number of clinical trials under the assumption that the true $p_{E}$ is $p_{E}^{H_{0}}$, the proportion of trials that conclude in favor of the experimental treatment (i.e., that lead to the rejection of the null hypothesis) represents an empirical evaluation of the type I error rate, while it represents an evaluation of the statistical power if the true value of $p_{E}$ used to simulate is $p_{E}^{H_{1}}$. Given *N*, *m* and a specified scenario $\left(p_{E}^{H_{0}},p_{E}^{H_{1}}\right)$, we calibrate the probability cut-off of the TS design by considering a dense set of possible values of *C*. For each value in the set, we simulate 100,000 trials assuming that the true $p_{E}$ is $p_{E}^{H_{0}}$, compute the empirical type I error probability and select the smallest value of *C* that controls the type I error rate at the nominal level 0.1. For the BOP2m design, a grid search is used to calibrate the tuning parameters λ and γ. For both of them, we consider a dense set of values in the interval (0, 1] and exhaustively enumerate all possible combinations. For each combination, we simulate 100,000 trials assuming that the true $p_{E}$ is $p_{E}^{H_{0}}$ and find the set of values of (λ, γ) that jointly yield a type I error rate lower than or equal to 0.1. Among the elements of this set of couples, we identify the one that maximizes the empirical statistical power obtained by simulating 100,000 trials under the assumption that the true $p_{E}$ is $p_{E}^{H_{1}}$. An analogous procedure is used to calibrate the probability thresholds of the LLm design. In this latter case, the grid search is performed by considering a dense set of values for $\theta_{T}$ and $\theta_{L}$ in the intervals (0.3, 0.99) and (0.01, 0.5), respectively.

Once the probability boundaries of the Bayesian design have been calibrated to have good frequentist operating characteristics, for each scenario we simulate 100,000 trials using different true values of $p_{E}$, that are $p_{E}^{H_{0}}$, $p_{E}^{H_{0}}$+0.1, $p_{E}^{H_{0}}$+0.2, and $p_{E}^{H_{0}}$+0.3. The performance of the Bayesian designs are evaluated by computing (i) the proportion of simulated trials where the null hypothesis is rejected (${\mathrm{PRH}}_{0}$), (ii) the probability of early termination (PET), empirically obtained through the proportion of simulated trials that terminate before reaching the maximum sample size, and (iii) the average of the actually achieved sample size (ASS). The obtained results are provided in Table 4 and Table 5 for different values of *N* and *m*, when δ=0.1. For each scenario used to calibrate the probability thresholds, we have highlighted in gray the operating characteristics under the null hypothesis. Thus, the values of ${\mathrm{PRH}}_{0}$ in gray represent the empirical type I error rate, that in all cases is no higher of 0.1 for construction. Generally, the BOP2m and the LLm designs show similar operating characteristics. When the true $p_{E}$ is larger than $p_{E}^{H_{0}}$, these two designs yield higher power levels and smaller risks of incorrectly terminating the trial early than the TS design. For instance, let us consider the scenario where $p_{E}^{H_{0}}=0.3$ and $p_{E}^{H_{1}}=0.5$. When N=40 and m=5, if the true response rate of *E* is 0.5, the empirical power is equal to 0.783, 0.886, and 0.875 for the TS, the BOP2m and the LLm designs, respectively. Moreover, the percentage of trials incorrectly terminated early is 21.2%, 8.8%, and 9.5% under the three designs, respectively. On the other hand, the TS design shows a higher probability of early termination under the null hypothesis. Furthermore, the TS design has a higher tendency to terminate the trial at the early stages and, as a consequence, it is characterized by lower expected values of the actually achieved sample size, which are especially desired under the null hypothesis. We can note that the LLm design generally yields the highest value of average sample size when $p_{E}$ is equal to $p_{E}^{H_{0}}$. This is because, when *n* is close to the maximum sample size, the predictive design typically requires higher observed response rates to let the trial proceed with respect to the other designs.

## 6. Discussion

The aim of this paper is to describe and compare Bayesian procedures used for futility monitoring of single-arm trials based on binary data. In this context, the Bayesian TS design [2] is very popular and has inspired several extensions and variations. We compare this design with the BOP2 design proposed by Zhou et al. [7] and the predictive design of Lee and Liu [11]. To have a fair comparison and to add flexibility to the decision rules, in line with Thall and Simon [2] we introduce a little change in these two latter designs to take into account the uncertainty in the response rate of the standard therapy.

The stopping boundaries of the Bayesian designs reflect the intent expressed by their futility rules. For instance, compared with the design of Thall and Simon, the BOP2 aims at introducing more relaxed rules at the early stages of the trial and, as a consequence, the minimum observed response rate required at the interim stage to avoid the termination of the trial increases as a function of the current sample size. Analogous considerations applies for the predictive design. The simulation results show that the statistical power is higher for the designs that define early stopping boundaries that take into account the ratio between the number of patients enrolled and the amount of future data. These designs also ensure lower probabilities of incorrectly terminating the trial early. However, they yield higher expected values of the actually achieved sample size under the assumption that the null hypothesis is true. We summarize below the main features of the three designs along with their advantageous characteristics shown in the simulation studies.
TS• Simpler and easier to implement
• Lower values of the ASS under $H_{0}$
 BOP2m• Takes into account the ratio between *n* and *N*
• Higher power and lower PET under $H_{1}$ if compared with TS
 LLm• Takes into account the number of remaining patients
• Resembles more closely the clinical decision-making process
• Higher power and lower PET under $H_{1}$ if compared with TS

Clearly, the decision rules compared are affected by the procedures used to calibrate the probability cut-offs of the designs. These adjustments are usually required by regulatory authorities to control the false positive rate of Bayesian procedures in a frequentist sense. Different calibration methods could be used, in order for instance to minimize the expected sample size under the null hypothesis, while controlling the type I error rate at a desired level.

Finally, let us notice that Thall and Simon [2] and Lee and Liu [11] also consider stopping rules for superiority of the experimental treatment. The same criteria could be implemented in the BOP2 design. However, in phase II single-arm trials investigators generally prefer to allow early stopping due to futility but not due to efficacy, because it is not considered unethical to continue the trial if the new treatment shows to be extremely effective [29]. This way of proceeding is consistent with the “ethical imperative for early termination” that characterizes the well-known two-stage scheme for single-arm phase II studies proposed by Simon [30] and that occurs when the treatment has unacceptably low efficacy. Instead, if the drug has substantial activity, there is interest in studying additional patients to better assess its safety and response. Many Bayesian two-stage designs exploit the Simon’s scheme to conduct a phase II study (see [19,26,31], among others). 

## Figures and Tables

**Table 1:** Stopping boundaries of the design by Thall and Simon [2], when N=40, $N_{min}=10$, δ=0.1, $\pi \left(p_{S}\right)=\mathrm{beta}\left(p_{S};\;63,\;94\right)$, $\pi \left(p_{E}\right)=\mathrm{beta}\left(p_{E};\;1.4,\;1.6\right)$ and the nominal level for the type I error rate is 0.1.

***n***	10	13	15	17	19	21	23	26	28	30	32	34	36	38	40
**$r_{n}$**	4	5	6	7	8	9	10	11	12	13	14	15	16	17	18

**Table 2:** Stopping boundaries of the modified version of the design by Zhou et al. [7], when N=40, $N_{min}=10$, δ=0.1, $\pi \left(p_{S}\right)=\mathrm{beta}\left(p_{S};\;63,\;94\right)$, $\pi \left(p_{E}\right)=\mathrm{beta}\left(p_{E};\;1.4,\;1.6\right)$ and the nominal level for the type I error rate is 0.1.

***n***	10	11	13	15	17	19	21	22	24	26	28	30	32	33	35	37	39	40
**$r_{n}$**	2	3	4	5	6	7	8	9	10	11	12	13	14	15	16	17	18	19

**Table 3:** Stopping boundaries of the modified version of the design by Lee and Liu [11], when N=40, $N_{min}=10$, δ=0.1, $\pi \left(p_{S}\right)=\mathrm{beta}\left(p_{S};\;63,\;94\right)$, $\pi \left(p_{E}\right)=\mathrm{beta}\left(p_{E};\;1.4,\;1.6\right)$ and the nominal level for the type I error rate is 0.1.

***n***	10	11	13	15	17	19	21	23	25	27	28	30	32	33	35	36	37	38	39	40
**$r_{n}$**	1	2	3	4	5	6	7	8	9	10	11	12	13	14	15	16	17	18	19	20

**Table 4:** Operating characteristics of the TS, BOP2m and LLm designs, when $N_{min}=10$, δ=0.1, N=40 and the type I error rate is controlled at the level 0.1. The lines in gray represent the operating characteristics under the null hypothesis.

Scenarios			Operating Characteristics When N=40 and m=1								
**Used to Calibrate**			**TS**			**BOP2m**			**LLm**		
$p_{E}^{H_{0}}$	**$p_{E}^{H_{1}}$**	**True $p_{E}$**	${\mathrm{PRH}}_{0}$	**PET**	**ASS**	${\mathrm{PRH}}_{0}$	**PET**	**ASS**	${\mathrm{PRH}}_{0}$	**PET**	**ASS**
0.2	0.4	0.2	0.098	0.902	16.56	0.099	0.901	20.88	0.084	0.868	28.11
		0.3	0.475	0.525	27.04	0.540	0.460	31.79	0.548	0.364	36.68
		0.4	0.819	0.181	35.33	0.894	0.106	38.01	0.923	0.053	39.55
		0.5	0.957	0.043	38.81	0.987	0.013	39.7	0.996	0.003	39.97
0.3	0.5	0.3	0.091	0.898	16.52	0.099	0.901	20.44	0.097	0.872	22.43
		0.4	0.428	0.561	26.04	0.483	0.517	30.53	0.502	0.447	32.48
		0.5	0.786	0.212	34.54	0.860	0.140	37.42	0.882	0.103	38.16
		0.6	0.952	0.048	38.70	0.984	0.016	39.64	0.988	0.011	39.73
0.4	0.6	0.4	0.093	0.900	15.97	0.094	0.888	20.57	0.072	0.903	25.56
		0.5	0.401	0.591	24.76	0.462	0.512	30.35	0.428	0.514	34.38
		0.6	0.762	0.236	33.64	0.860	0.132	37.51	0.864	0.110	39.01
		0.7	0.943	0.057	38.37	0.987	0.013	39.72	0.992	0.006	39.94
0.5	0.7	0.5	0.093	0.899	15.80	0.094	0.893	20.14	0.074	0.904	24.90
		0.6	0.405	0.587	24.69	0.463	0.516	30.09	0.433	0.519	34.06
		0.7	0.777	0.222	33.95	0.872	0.123	37.62	0.879	0.102	39.07
		0.8	0.958	0.042	38.80	0.992	0.008	39.80	0.996	0.003	39.97
**Scenarios**			**Operating Characteristics When N=40 and m=5**								
**Used to Calibrate**			**TS**			**BOP2m**			**LLm**		
**$p_{E}^{H_{0}}$**	**$p_{E}^{H_{1}}$**	**True $p_{E}$**	${\mathrm{PRH}}_{0}$	**PET**	**ASS**	${\mathrm{PRH}}_{0}$	**PET**	**ASS**	${\mathrm{PRH}}_{0}$	**PET**	**ASS**
0.2	0.4	0.2	0.093	0.869	18.41	0.070	0.881	22.28	0.086	0.626	30.99
		0.3	0.499	0.467	29.10	0.487	0.436	32.53	0.553	0.157	37.99
		0.4	0.852	0.142	36.60	0.883	0.098	38.31	0.926	0.016	39.77
		0.5	0.973	0.027	39.31	0.989	0.010	39.80	0.996	0.001	39.99
0.3	0.5	0.3	0.098	0.878	16.92	0.096	0.826	23.89	0.097	0.815	22.70
		0.4	0.442	0.534	26.28	0.501	0.397	33.23	0.497	0.388	32.42
		0.5	0.783	0.212	34.19	0.886	0.088	38.51	0.875	0.095	37.90
		0.6	0.941	0.058	38.30	0.991	0.008	39.85	0.984	0.015	39.60
0.4	0.6	0.4	0.097	0.890	16.69	0.097	0.856	21.93	0.073	0.727	28.22
		0.5	0.415	0.572	25.58	0.469	0.470	31.22	0.432	0.286	36.04
		0.6	0.776	0.222	34.09	0.865	0.119	37.80	0.868	0.041	39.42
		0.7	0.947	0.053	38.46	0.989	0.011	39.78	0.993	0.002	39.97
0.5	0.7	0.5	0.097	0.885	16.10	0.096	0.873	21.59	0.075	0.766	28.10
		0.6	0.408	0.575	24.85	0.469	0.489	31.06	0.439	0.319	35.98
		0.7	0.775	0.222	33.96	0.877	0.114	37.93	0.883	0.042	39.51
		0.8	0.958	0.042	38.80	0.993	0.007	39.84	0.997	0.001	39.99

**Table 5:** Operating characteristics of the TS, BOP2m and LLm designs, when $N_{min}=10$, δ=0.1, N=80 and the type I error rate is controlled at the level 0.1. The lines in gray represent the operating characteristics under the null hypothesis.

Scenarios			Operating Characteristics When N=80 and m=1								
**Used to Calibrate**			**TS**			**BOP2m**			**LLm**		
**$p_{E}^{H_{0}}$**	**$p_{E}^{H_{1}}$**	**True $p_{E}$**	${\mathrm{PRH}}_{0}$	**PET**	**ASS**	${\mathrm{PRH}}_{0}$	**PET**	**ASS**	${\mathrm{PRH}}_{0}$	**PET**	**ASS**
0.2	0.4	0.2	0.099	0.901	29.02	0.095	0.905	38.91	0.065	0.910	50.73
		0.3	0.643	0.357	59.85	0.723	0.277	69.21	0.709	0.241	75.26
		0.4	0.926	0.074	75.22	0.979	0.021	78.93	0.989	0.008	79.75
		0.5	0.987	0.013	79.09	0.999	0.001	79.92	1.000	0.000	79.99
0.3	0.5	0.3	0.100	0.900	27.64	0.099	0.901	37.47	0.087	0.888	51.87
		0.4	0.579	0.421	55.80	0.664	0.336	66.29	0.696	0.262	74.73
		0.5	0.900	0.100	73.52	0.967	0.033	78.35	0.987	0.011	79.68
		0.6	0.983	0.017	78.81	0.998	0.002	79.89	1.000	0.000	79.98
0.4	0.6	0.4	0.099	0.901	26.68	0.096	0.898	37.57	0.100	0.878	51.55
		0.5	0.548	0.452	53.46	0.639	0.355	65.59	0.693	0.273	74.20
		0.6	0.887	0.113	72.66	0.967	0.033	78.33	0.987	0.011	79.64
		0.7	0.982	0.018	78.81	0.999	0.001	79.91	1.000	0.000	79.98
0.5	0.7	0.5	0.098	0.902	26.26	0.100	0.893	36.87	0.098	0.884	46.84
		0.6	0.547	0.453	53.23	0.649	0.344	65.51	0.688	0.286	72.22
		0.7	0.896	0.104	73.17	0.973	0.027	78.58	0.987	0.012	79.48
		0.8	0.988	0.012	79.20	0.999	0.001	79.95	1.000	0.000	79.98
**Scenarios**			**Operating Characteristics When N=80 and m=5**								
**Used to Calibrate**			**TS**			**BOP2m**			**LLm**		
**$p_{E}^{H_{0}}$**	**$p_{E}^{H_{1}}$**	**True $p_{E}$**	${\mathrm{PRH}}_{0}$	**PET**	**ASS**	${\mathrm{PRH}}_{0}$	**PET**	**ASS**	${\mathrm{PRH}}_{0}$	**PET**	**ASS**
0.2	0.4	0.2	0.098	0.895	29.59	0.082	0.887	40.49	0.066	0.794	55.39
		0.3	0.645	0.352	60.38	0.713	0.255	70.04	0.717	0.129	76.89
		0.4	0.929	0.071	75.44	0.979	0.020	78.96	0.991	0.003	79.89
		0.5	0.987	0.013	79.11	0.999	0.001	79.91	1.000	0.000	80.00
0.3	0.5	0.3	0.093	0.894	28.38	0.098	0.882	42.09	0.088	0.796	55.70
		0.4	0.577	0.415	56.26	0.687	0.293	69.43	0.703	0.168	76.08
		0.5	0.900	0.100	73.54	0.977	0.022	78.99	0.988	0.006	79.80
		0.6	0.982	0.018	78.78	0.999	0.001	79.95	1.000	0.000	79.99
0.4	0.6	0.4	0.099	0.885	29.00	0.097	0.893	39.49	0.099	0.815	51.69
		0.5	0.571	0.420	56.22	0.644	0.346	66.67	0.690	0.212	74.20
		0.6	0.907	0.093	74.10	0.970	0.029	78.60	0.986	0.009	79.62
		0.7	0.987	0.013	79.12	0.999	0.001	79.94	1.000	0.000	79.98
0.5	0.7	0.5	0.093	0.896	27.21	0.082	0.880	39.39	0.099	0.800	48.58
		0.6	0.549	0.444	54.19	0.631	0.326	67.09	0.691	0.205	72.98
		0.7	0.904	0.096	73.82	0.977	0.022	78.98	0.988	0.009	79.54
		0.8	0.990	0.010	79.33	1.000	0.000	79.98	1.000	0.000	79.99

## Data Availability

Not applicable.

## Abbreviations

TS	Design due to Thall and Simon [2]
BOP2m	Modified version of the BOP2 design due to Zhou et al. [7] to account for uncertainty in $p_{S}$
LLm	Modified version of the design of Lee and Liu [11] to account for uncertainty in $p_{S}$
${\mathrm{PRH}}_{0}$	Proportion of simulated trials where the null hypothesis is rejected
PET	Probability of early termination
ASS	Average of the actually achieved sample size
